# Evaluating the Ectasia Risk Score System in Cancelled Laser In Situ Keratomileusis Candidates

**DOI:** 10.18502/jovr.v15i4.7788

**Published:** 2020-10-25

**Authors:** Mehrdad Mohamadpour, Masoud Khorrami-Nejad, Mohammad Yaser Kiarudi, Keivan Khosravi

**Affiliations:** ^1^Translational Ophthalmology Research Center, Farabi Eye Hospital, Tehran University of Medical Sciences, Tehran, Iran; ^2^School of Rehabilitation, Shahid Beheshti University of Medical Sciences, Tehran, Iran

**Keywords:** Dexamethasone Implant, Diabetic Macular Edema

## Abstract

**Purpose:**

To evaluate the ectasia risk score system in cancelled laser in situ keratomileusis (LASIK) candidates at an academic hospital.

**Methods:**

LASIK candidates who had been cancelled by a surgeon considering the patient age, preoperative central corneal thickness, residual stromal bed thickness, or preoperative manifest refraction spherical equivalent were retrospectively reviewed, and their Randleman ectasia risk score system score was calculated.

**Results:**

The mean ectasia score of 194 eyes (97 patients) was 4.5 ± 2.67; 40 (20.6%), 46 (23.7%), and 108 (55.7%) eyes were classified as low-, moderate-, and high-risk eyes, respectively. The topography was abnormal in 69% of the patients. The mean manifest refraction spherical equivalent, central corneal thickness, and estimated residual stromal bed thickness were 4 (+0.50 to –15.50) diopters, 520 (439 to 608) µm, and 312.38 (61.5 to 424.12) µm, respectively. The main cause of cancellation in low- and moderate-risk patients (86 eyes) was the presence of unstable refractive error in the past year.

**Conclusion:**

Although promising, some other criteria, such as stable refraction, should be added to this scoring system to achieve greater practicality since a main cause of cancelling LASIK candidates in this study was the presence of unstable refraction.

##  INTRODUCTION

Ectasia, progressive steepening, and thinning of the cornea are uncommon but serious complications of excimer laser corneal refractive surgery that reduce uncorrected and often corrected distance visual acuity (CDVA). They occur commonly after laser in situ keratomileusis (LASIK) and infrequently after photorefractive keratectomy (PRK).^[1]^ Because of the significant medical and legal consequences of this complication, studies have focused on this issue.^[2,3,4,5,6,7,8,9]^

Ectatic changes can occur as early as one week after LASIK or be delayed for up to several years after the initial procedure.^[10]^ Histologic findings suggest that post-LASIK keratectasia results in collagen fibril thinning and a decreased interfibrillar distance within the residual stromal bed (RSB).^[11]^

Clinical and topographical findings of ectasia are often indistinguishable from those of keratoconus.^[12]^ To date, no method can definitively diagnose patients with ectasia. A practical task for clinicians is to improve the sensitivity of screening methods for identifying patients with mild keratoconus to prevent iatrogenic keratectasia. Randleman et al^[1]^ developed a scoring system to evaluate the risk of ectasia after LASIK considering some parameters – such as preoperative topography, RSB thickness, age, preoperative corneal thickness, and degree of myopia – to better identify patients with a high risk for ectasia. They concluded that no single characteristic identifies all at-risk patients. Instead, various factors should be considered in a weighted fashion. For all patients, special emphasis should be placed on the topographic pattern, and factors in addition to the inferior–superior value or computer-generated indices should be considered in the screening. For young patients, heightened scrutiny is warranted, and other aspects of their preoperative evaluation, particularly topographic patterns and refractive stability, should be within normal limits. The intraoperative corneal thickness should be measured if the variability of flap thickness is likely to put a patient at risk for ectasia. In the initial series, this scoring system showed a high sensitivity and specificity. Subsequently, another study validated it and noted the ectasia risk score system (ERSS) to be a valid and effective method for detecting the eyes with ectasia after LASIK.^[13]^

This study was aimed at retrospectively evaluating the ERSS score in candidates for refractive surgery who were cancelled by a surgeon. We calculated the risk score of cancelled LASIK candidates to evaluate the efficacy of ERSS for the preoperative screening.

##  METHODS

Medical records of candidates for refractive surgery who had been cancelled by a surgeon (MM) at Farabi Eye Hospital, Tehran University of Medical Sciences, Tehran, Iran, between 2011 and 2015 were reviewed. Causes of cancellation of these candidates were based on conventional and individual criteria, such as unstable refraction, defined as an increase in the spherical or cylindrical refractive error >0.5 diopters (D) in the past year, age <9 years, or suspected or abnormal topographical patterns, such as inferior steepening >1.4 D, central corneal thickness <480 µm, RSB <250 µm, or CDVA <20/30. The exclusion criteria were a history of refractive or cataract surgery, keratoconus, collagen vascular disease, and diabetic retinopathy.

Data of all patients, including the age, sex, preoperative manifest refraction spherical equivalent, central corneal thickness, topographic pattern, and predicted RSB thickness were evaluated, and their Randleman ectasia risk factor score was calculated. Based on the Randleman ERSS, as described previously,^[1]^ a cumulative ectasia risk score was calculated for all patients. Central corneal and RSB thicknesses were measured using a Pentacam topographer (Oculus, Wentzler, Germany). Regarding the cumulative points, risk categories were as follows: 0–2 points, low risk; 3 points, moderate risk; and 4 points, high risk.

##  RESULTS

A total of 97 patients (194 eyes) were included in this study, with the mean age of 26.4 years (range: 18–50 years). Sex distribution was approximately equal (48 women and 49 men).

According to the Randleman ERSS for preoperative refractive surgery, 40 (20.6%), 46 (23.7%), and 108 (55.7%) eyes had low-, moderate-, and high-risk scores, respectively. The mean score was 4.5 ± 2.67. The mean manifest refraction spherical equivalent, central corneal thickness, and estimated RSB thickness were 4 (+0.5 to –15.5) D, 520 (439 to 608) µm, and 312.38 (61.5 to 424.12) µm, respectively (Table 1). Refractive astigmatism ranged from 0.5 to 6.25 D. Corneal astigmatism ranged from 0.04 to 4.90 D. Internal astigmatism ranged from 0 to 2.25 D. Unstable refraction was found in 86 (44.3%) eyes.

**Table 1:** Randleman ectasia risk score system for identifying eyes at high risk of developing ectasia after laser in situ keratomileusis


**Parameter**	**Points**				
	**4**	**3**	**2**	**1**	**0**
**Topography**	**Abnormal topography**	**INF steep/SRA**	**ABT**	**Normal/SBT**
RSB (µ)	<240	240 to 259	260 to 279	280 to 299	≥300
Age (yrs)	18 to 21	22 to 25	26 to 29	≥30
CT (µ)	<450	451 to 480	481 to 510	≥510
MRSD (D)	>–14	>–12 to –14	>–10 to –12	>–8 to –10	>–8 or less
Low risk (0–2 points), moderate risk (3 points), high risk (≥4 points) ABT, asymmetric bowtie; CT, corneal thickness; D, diopters; INF steep, inferior steepening pattern; MRSE, manifest refraction spherical equivalent; RSB, residual stromal bed thickness; SBT, symmetric bowtie; SRA, skewed radial axis; Yrs, years					

**Table 2:** Characteristics of 97 patients (194 eyes)


**Features**	**Mean (range)**
Age (years)	26.44 (18 to 50)
Gender Male Female	49 (50.5%) 48 (49.5%)
MRSE (D)	4 (0.5 to 15.5)
CCT (µ)	520 (439 to 608)
RSB (µ)	312.38 (61.5 to 424.12)
CCT, central corneal thickness; D, diopters; MRSE, manifest refraction spherical equivalent; RSB, residual stromal bed thickness	

**Table 3:** Topographic patterns of 194 eyes of 97 patients


**Topographic pattern**	**Number (Percentage)**
Normal/symmetrical	65 (33.51)
Asymmetric bowtie	87 (44.84)
INF steep/SRA	39 (20.11)
Abnormal	3 (1.54)
INF Steep/SRA, topographic pattern with inferior steepening and/or a skewed radial axis	

Table 2 shows the topographic characteristics of the patients. The symmetrical (normal) pattern, asymmetrical bow tie, inferior steepening or skewed radial axis, and other abnormal topographic (pellucid) patterns were seen in 65 (33.51%), 87 (44.84%), 39 (20.1%), and 3 (1.54%) eyes, respectively.

##  DISCUSSION

In our study, a significant number of patients (86 eyes, 43.7%) who were not scheduled for LASIK due to unstable refraction in the past year had a low or moderate ERSS score (1–3). Therefore, this method, which is applied for categorizing and assessing the risk of ectasia, should be modified in consideration of unstable refractive errors.

Reports on ERSS are incongruent.^[13,14,15,16]^ Previously, Randelman et al stated that this system, which was developed from case reports of ectasia, was more sensitive compared to traditional screening strategies. In a subsequent study, Randelman et al validated their risk scoring system by applying it on 50 previously unpublished ectasia cases matched to 50 normal eyes. The sensitivity and specificity of their scoring system for the initial and comparison populations were 91% and 92%, respectively.^[13]^

Binder et al^[15]^ retrospectively reviewed a surgeon's LASIK database to assess Randleman ectasia risk scores in eyes with normal preoperative topography. Risk scores of 5 or more, 4 or more, and 3 or more were found in 35 (2.1%), 92 (5.4%), and 208 (12.2%) eyes with a normal topography, respectively. None of these eyes developed ectasia. In their evaluation, three eyes of two patients in the entire database of 9,813 myopic eyes with variable follow-up periods developed ectasia. They noted that with this risk score system, 5.4% of eyes with 4 or more points would have been eliminated from LASIK surgery, and an additional 6.8% of eyes with a score of 3 indicating a “moderate risk” would have required the surgeon to exercise caution. Therefore, they concluded that in the eyes with normal preoperative topographies, this scoring system may not accurately predict an increased risk for developing postoperative LASIK ectasia. This is consistent with our findings of a significant number of patients at a high risk whom the surgeon cancelled, while the risk score was low or moderate with ERSS.

Chan et al^[14]^ retrospectively evaluated ERSS in 36 eyes with post-LASIK ectasia. A low risk was seen in 25% of eyes. They reported the sensitivity of this method to be only 56% and concluded that ERSS can miss a significant proportion of patients at risk of ectasia. Randleman et al^[1]^ reported that unstable refractions may predict corneal ectasia after refractive surgery. This study suggests that other criteria, such as stable refraction, should be added to this scoring system for increased practicality since a main cause of cancelled LASIK candidates in this study was unstable refraction.

Hodge et al^[16]^ presented a case of unilateral keratectasia in a laser refractive surgery patient. LASIK was performed in the first eye, but because of the difficulty in lifting the femtosecond-created cap in the fellow eye, PRK was performed in that eye. In their report, the eyes had no risk factors for keratectasia and identical low Randleman ERSS scores. According to them, the procedure of elevating the cap had a weakening effect.

Consistent with Sorkin et al's study,^[17]^ a highly prevalent risk factor in our study was suspected and abnormal topographic patterns were found in approximately 64% of patients. This is consistent with former reports. Nearly 50% of ectasia cases in Randleman et al's study and 69% in Chan et al's study had abnormal topographies. ERSS relies exclusively on Placido-based images. Recent topographic systems apply other corneal imaging, including Orbscan II and Pentacam imaging. The limitations of ERSS include starting keratoconus from the posterior portion and the lack of assessment of posterior elevation.

Another factor not included in our study for ERSS was unstable refraction. Randleman et al^[1]^ considered refractive instability in borderline cases that could increase the risk of ectasia. We defined it as a spherical or cylindrical refractive error >0.5 D in the past year and found it in 86 (44.3%) eyes. This factor may be paramount for an effective refractive surgical screening.

Dawson et al evaluated the histopathology and ultrastructure of the corneas developing ectasia after LASIK or PRK and found no significant differences in any of the measurements in the LASIK flap or interface wound; however, the ultrastructure of RSB significantly differed between the ectatic and non-ectatic regions. The primary effect of the biomechanical failure process involves the RSB (42% thickness reduction) compared to the LASIK flap or hypocellular primitive stromal scar.^[18]^ In this regard, preservation of at least a 250-μm thickness in the central stromal bed after ablation is important for the prevention of ectasia,^[19]^although the suggested range of minimum RSB varies from 200 to 318 μm.^[20,21]^ However, keratectasia can occur after LASIK with an RSB thickness of >250 μm.^[22]^ In our study, the mean RSB thickness of cancelled patients was 312 μm above the widely accepted 250 μm. Moreover, in Chan et al's study, the average RSB was >250 μm.^[14]^ Therefore, it cannot be an isolated risk factor.

We suggest considering refractive stability as a factor with a significant weight in a modified scoring system for the preoperative screening for ablative refractive surgery. Validation of other possible values, such as the degree of astigmatism, between eye topographic asymmetry, and family history of keratoconus should be evaluated. Moreover, studies should include a control group comprising the eyes that have undergone LASIK at the same interval. In conclusion, Randleman ERSS, although promising, is yet to be validated with a population of ectasia cases and controls.

##  Financial Support and Sponsorship

None.

##  Conflicts of Interest

There are no conflicts of interest.
